# Altered Nasal Microbiota Composition Associated with Development of Polyserositis by *Mycoplasma hyorhinis*

**DOI:** 10.3390/pathogens10050603

**Published:** 2021-05-14

**Authors:** Miguel Blanco-Fuertes, Florencia Correa-Fiz, Lorenzo Fraile, Marina Sibila, Virginia Aragon

**Affiliations:** 1 IRTA, Centre de Recerca en Sanitat Animal (CReSA, IRTA-UAB), Campus de la Universitat Autònoma de Barcelona, 08193 Bellaterra, Spain; miguel.blanco@irta.cat (M.B.-F.); marina.sibila@irta.cat (M.S.); virginia.aragon@irta.cat (V.A.); 2 OIE Collaborating Centre for the Research and Control of Emerging and Re-Emerging Swine Diseases in Europe (IRTA-CReSA), 08193 Bellaterra, Spain; 3 Departamento de Ciencia Animal, Escuela Técnica Superior de Ingeniería Agraria (ETSEA), Universidad de Lleida, 25198 Lleida, Spain; lorenzo.fraile@ca.udl.cat

**Keywords:** porcine polyserositis, nasal microbiota, *Mycoplasma hyorhinis*, microbial diversity, 16S rRNA gene, Glässer’s disease

## Abstract

Fibrinous polyserositis in swine farming is a common pathological finding in nursery animals. The differential diagnosis of this finding should include *Glaesserella parasuis* (aetiological agent of Glässer’s disease) and *Mycoplasma hyorhinis*, among others. These microorganisms are early colonizers of the upper respiratory tract of piglets. The composition of the nasal microbiota at weaning was shown to constitute a predisposing factor for the development of Glässer’s disease. Here, we unravel the role of the nasal microbiota in the subsequent systemic infection by *M. hyorhinis*, and the similarities and differences with Glässer’s disease. Nasal samples from farms with recurrent problems with polyserositis associated with *M. hyorhinis* (MH) or Glässer’s disease (GD) were included in this study, together with healthy control farms (HC). Nasal swabs were taken from piglets in MH farms at weaning, before the onset of the clinical outbreaks, and were submitted to 16S rRNA gene amplicon sequencing (V3–V4 region). These sequences were analyzed together with sequences from similar samples previously obtained in GD and HC farms. Animals from farms with disease (MH and GD) had a nasal microbiota with lower diversity than those from the HC farms. However, the composition of the nasal microbiota of the piglets from these disease farms was different, suggesting that divergent microbiota imbalances may predispose the animals to the two systemic infections. We also found variants of the pathogens that were associated with the farms with the corresponding disease, highlighting the importance of studying the microbiome at strain-level resolution.

## 1. Introduction

The microbiota is defined as the community of microorganisms found in the surface of a tissue, which represents their usual ecological niche [1]. Several studies have found an association between the microbiota composition and phenotypic features from the host, such as body weight, health status and disease onset, among others [2]. In animals, and in particular swine, the gut microbiota has been deeply studied in contrast to other body sites. The porcine intestinal microbiota influences several production parameters like body weight [3] and feed efficiency [4]. The alterations in its composition can affect the translocation of metabolites across a disrupted intestinal barrier, which in turn could promote metabolic pathologies in various organs, such as liver and adipose tissue [4]. Although to a lesser extent, the nasal microbiota has also been associated with the development of diseases [5]. In swine, the nasal microbiota has been suggested as a predisposing factor for the development of Glässer’s disease, a systemic infection caused by *Glaesserella* (*Haemophilus*) *parasuis* and characterized by fibrinous polyserositis [6].

Polyserositis is a common finding in necropsies during the post-weaning period. This pathology is not only caused by *G. parasuis*, but also by other bacteria, such as *Mycoplasma hyorhinis* [7]. These microorganisms are considered early colonizers of the respiratory tract, as they are part of the bacterial communities that conform the normal microbiota of the nasal cavity of piglets early in life [6,8,9,10]. How these etiological agents switch from members of the normal microbiota to disseminate and induce systemic infection is the key to understand the pathogenesis of these diseases. A balance between colonization by these potential pathogens and the host is established during the early stages of the piglets’ life [11] where the immune system plays a crucial role to establish this balance and maintain health. Recently, the role of particular taxa from the piglet microbiota has been shown in the polyserositis caused by *G. parasuis* where *Lactobacillus* and *Prevotella* were associated with health, while *Moraxella*, *Haemophilus* (*Glaesserella*) and *Streptococcus* were associated with Glässer’s disease [6,12]. Similarly, differences in the oropharyngeal microbiota have been associated with the development of respiratory diseases in pigs [13]. Higher relative abundance of the *Moraxella* genus was associated with respiratory pathology and *Lactobacillus* was associated with healthy animals [13,14].

Here, we compare the piglet nasal microbiota composition at weaning, in farms with and without polyserositis cases caused by *M. hyorhinis*. Furthermore, we compare the nasal microbiota composition from a *M. hyorhinis*-affected farm, with data from previously characterized farms with recurrent outbreaks of Glässer’s disease, and divergent changes from health between both pathological scenarios.

## 2. Results

### 2.1. Alpha Diversity Is Significantly Reduced in the Nasal Microbiota of Piglets from Farms with Polyserositis

Farms with post-weaning polyserositis caused by *M. hyorhinis* (MH group) or *G. parasuis* (GD group), together with healthy control (HC group) farms, were included in this study (Table 1). Nasal samples were obtained from piglets at weaning and the nasal microbiota was determined by 16S rRNA gene sequencing. We obtained a total of 33,012,373 reads from the sequencing data of 74 nasal samples. The minimum number of reads in a sample was 70,970 and the maximum was 121,478. After quality-control, denoising and chimera removal, a total of 5,061,172 sequences were included in the posterior analysis.

**Figure 1:**
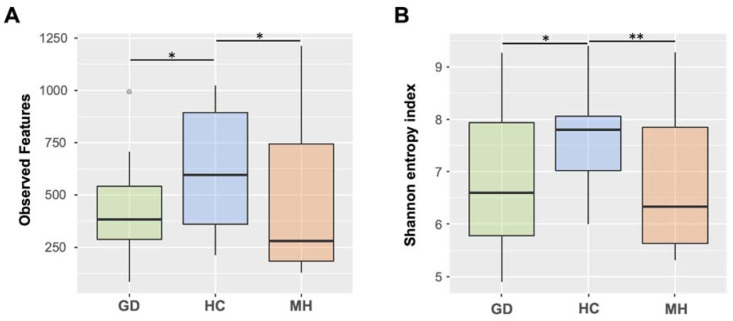
Boxplots representing median and interquartile ranges of alpha diversity estimated measuring the observed features (**A**) or Shannon’s index (**B**) in nasal microbiota from piglets at weaning from healthy control farms (HC) and farms with polyserositis in the nursery caused by *Mycoplasma hyorhinis* (MH) or caused by *Glaesserella parasuis* (Glässer’s disease, GD). Outlier is indicated with a grey circle on the plot. Error bars are standard deviation. * means *p* < 0.05; ** means *p* ≤ 0.01.

The feature table was rarefied to 6,545 sequences per sample and alpha diversity was estimated using different metrics. Microbial richness was assessed by calculating the observed features, which gives the count of the different amplicon sequence variants (ASVs) in rarefied samples. We found a total of 25,993 ASVs, with significant differences in richness among the three farm groups (Kruskal-Wallis; *p* = 0.027). Richness was significantly higher in the HC farms when compared with the farms with polyserositis MH and GD individually (Figure 1A). Chao index showed the same trend and detected the same differences between the groups (not shown). Alpha diversity was additionally assessed by Shannon index (Figure 1B), which considers both the richness and the evenness of the samples, and also detected differences among the three groups (Kruskal-Wallis; *p* = 0.017). Farms with disease showed lower diversity than HC group when compared individually (HC vs MH and HC vs GD, *p* = 0.01 in both cases; Figure 1B).

### 2.2. Piglets from Farms with Polyserositis by M. hyorhinis Showed Significatively Different Nasal Microbiota Composition Compared to Piglets from HC and GD Farms

**Figure 2:**
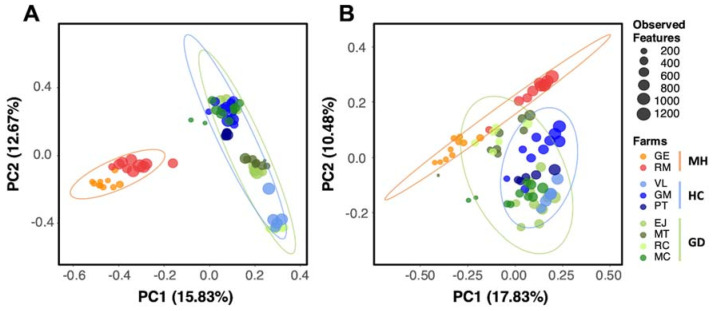
Beta diversity analysis on Bray Curtis (**A**) and unweighted Unifrac distances (**B**) of the nasal microbiota of weaning piglets. Each circle represents the microbial composition of each sample and the size of the circles is proportional to the number of Observed Features as indicated in the legend. Healthy control farms (HC) are depicted in blue, while farms with polyserositis caused by *M. hyorhinis* (MH) are in orange and *G. parasuis* (GD) in green palettes. Ellipses are calculated with the Euclidean distances of the grouped samples.

The differences in composition (beta diversity) of the nasal microbial communities among the different study groups were explored using Principal Coordinates Analysis (PCoA). The beta diversity analysis was done using Bray Curtis distance to measure the compositional dissimilarity quantitatively between different groups. The PCoA plot showed a more similar nasal microbiota composition between animals from GD and HC farms compared with the MH farms (Figure 2A). Although strong clustering was observed when the samples were compared considering the farm of origin (R^2^ = 47% estimated by Adonis function), the differences among the three groups of farms were explained by 17.3% (R^2^, *p* = 0.001, 999 permutations). Moreover, to understand the qualitative dissimilarities between groups the Jaccard distances were calculated. The Adonis function on this qualitative approach retrieved values for R^2^ of 26.12% and 9.52% for farm or group clustering, respectively (Supplementary Figure S1A).

We also measured the qualitative (unweighted) and quantitative (weighted) measures of community dissimilarity incorporating the phylogenetic relationships between features (Unifrac). The percentage explained by the clustering of these groups was higher in the unweighted analysis (R^2^ = 12.07% Adonis, *p* = 0.01; Figure 2B) than in the weighted analysis (R^2^ = 10.04% Adonis, *p* < 0.01; Supplementary Figure S1B). The farm of origin had also a strong effect in the clustering (R^2^ = 36.6% Adonis, *p* = 0.01).

### 2.3. Taxonomic Assignment of the ASVs

Taxa assignment was done by Naive Bayes classification algorithm using the GreenGenes 13.8 16S gene database. Taxonomy was assigned to a total of 25,993 ASVs. The percentage of unassigned ASVs was increasing from phylum, with 0.96% of unassigned taxa, to lower taxonomic levels until species where 89.44% were found.

At phylum level, more than the 80% of the relative abundance were shared between three phyla, *Proteobacteria*, *Bacteroidetes* and *Firmicutes*. *Proteobacteria* was the most relative abundant phylum in the disease groups, while *Bacteroidetes* and *Firmicutes* were the most relatively abundant in the healthy group. *Gammaproteobacteria* was the most relative abundant bacterial class of the microbiota composition in the disease groups (MH and GD), while *Clostridia* was the most abundant in the healthy one. Indeed, *Clostridiales* and *Pseudomonales* were the most represented orders in the HC and disease groups, respectively.

At the family level, the most abundant were *Moraxellaceae*, *Weeksellaceae*, *Pasteurellaceae*, *Ruminococcaceae*, *Lachnospiraceae*, *Prevotellaceae*, *Muribaculaceae (S24-7)*, *Streptococcaceae* and *Mycoplasmataceae*. *Moraxellaceae* was the most relative abundant family in all groups, with 17.69% in the HC group, 26.09% in GD and 32.30% in the MH group.

The most abundant genus in all groups was *Bergeyella*, with 14.45% in the HC group, 17.26% in GD and 20.85% in the MH group. *Enhydrobacter* was the second most abundant genus in HC and MH group with 6.30% and 18.19%, respectively. *Moraxella* was the second most abundant genus in GD (9.73%) and the third one in the MH group with the same percentage of 9.73. Finally, an unclassified genus from *Moraxellaceae* was the third most abundant in HC (5.76%) and GD (9.39%).

**Figure 3:**
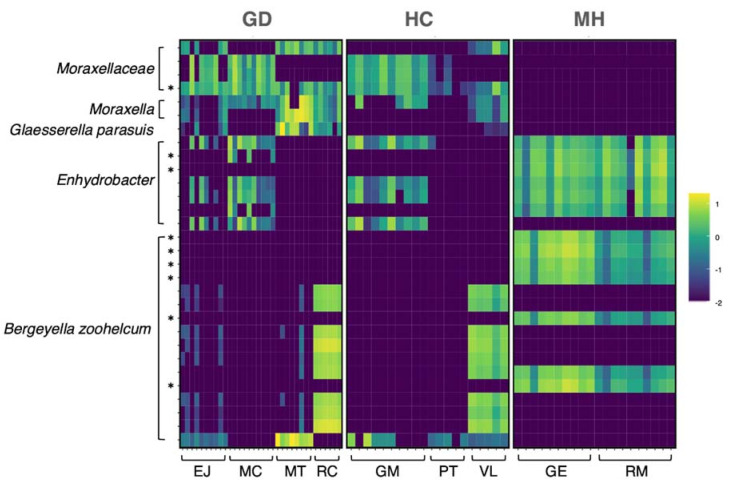
Relative abundance in the nasal microbiota of weaning piglets of the top 30 most abundant ASVs among all the groups in log10 scale. The X axis (bottom) shows the distribution of each sample by farm and the Y axis represents the top 30 most abundant ASVs grouped by the taxa assignment. Top labels correspond to the study groups: Glässer’s disease (GD), healthy control (HC) and *M. hyorhinis* (MH) farms. * Asterisks mark the ASVs detected as statistically different in the differential abundance analysis (ANCOM) among the three study groups (GD, MH, HC).

**Figure 4:**
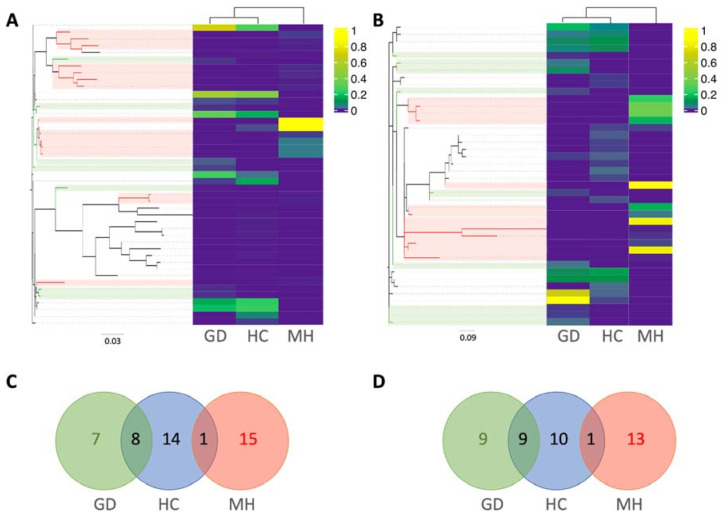
Relative abundance of *M. hyorhinis* (**A**) and *G. parasuis* (**B**) amplicon sequence variants (ASVs) in the nasal microbiota of weaning piglets from farms with Glässer’s disease (GD), healthy controls (HC) or farms with polyserositis caused by *M. hyorhinis* (MH). The phylogenetic relationship of ASVs from each pathogen is represented in Maximum likelihood trees. Branches from ASVs found exclusively in GD farms are colored in green while the ones found only the MH group are colored in red. Venn diagrams representing the distribution of *M. hyorhinis* (**C**) and *G. parasuis* (**D**) ASVs in the different groups were plotted following the same color pattern.

The 30 most abundant ASVs across all the samples belonged to *Moraxellaceae* (n = 4), *Moraxella* (n = 2), *Enhydrobacter* (n = 7), *G.* (*Haemophilus*) *parasuis* (n = 1) and *Bergeyella zoohelcum* (n = 17), which were distinctly distributed depending on the group (Figure 3). If we focus on the ASVs from *G. parasuis* and *M. hyorhinis*, the aethiological agents of the polyserositis observed in the farms, 45 ASVs were classified as *M. hyorhinis* (Figure 4A) and 42 as *G. parasuis* (Figure 4B). Curiously, the ASVs from these pathogens were not shared among the groups, but they were shared among different farms from the same group (Figure 4C,D). Interestingly, the amplicon 16S sequences amplified from the isolated clinical samples were highly similar (99–100%) to the two most abundant *M. hyorhinis* ASVs in the MH group clustering together into the phylogenetic tree (Figure 4A, strong yellow).

#### Differential Abundance Analysis

To find out taxa differentially abundant among different groups we used the Analysis of composition of Microbiome (ANCOM) method. When the nasal composition from the three groups was compared, 144 differential ASVs were detected (Supplementary Table S7). The most significant ASVs belonged to *Moraxellaceae*, *Bergeyella zoohelcum*, *Enhydrobacter*, *Moraxella* and *Rothia nasimurium*. Remarkably, most of these ASVs detected as differential were present with a high relative abundance in the MH group when compared with the other two groups. Two of the high-abundant ASVs from MH group were classified as *M. hyorhinis*, depicted in Figure 4A in bright yellow. On the other hand, one ASV was classified as *G. parasuis* but was only present in the MH group. Moreover, nine of the differentially abundant ASVs were found among the top 30 most abundant among all the groups globally, which are indicated with an asterisk in Figure 3.

To explore if there were ASVs associated to the health status, we analyzed groups MH and GD joined as a “disease group”, against control (HC). Only eight features appeared as differentially abundant between these two groups, including three from *Prevotella* genus and one from *Streptococcus alactolyticus*, all in higher abundance in healthy farms (Supplementary Table S1). Additionally, when GD group was compared to the HC, only one ASVs from the *Lachnospiraceae* family was detected as differentially abundant. However, when MH was compared to HC, 67 ASVs were detected (Supplementary Table S1), where the ten most significant ASVs included seven classified as *B. zoohelcum*, two as *Enhydrobacter* and one as *Moraxellaceae* family. These latter ASV was the only one (from these ten) associated with the control farms (absent in MH farms), since the seven ASVs from *B. zoohelcum* were only present in the MH group, (with relative abundances ranging from 1.73% to 3.51%) and the two ASVs classified as *Enhydrobacter* were absent in HC farms (Supplementary Table S1).

The differences observed at the ASVs level were not reflected in the differential analysis (ANCOM) derived from the collapsed ASVs table at the different taxonomic levels (Supplementary Tables S2–S7).

## 3. Discussion

Culture-independent studies for interpreting the swine microbiome in disease infections can be challenging, especially if the study aims to elucidate the differences in the microbiome before the onset of the clinical signs as a predisposing factor. Here, we studied the relationship between the nasal microbiota and the later development of the systemic infection by the early colonizers of the respiratory tract, *M. hyorhinis* and *G. parasuis*. We found lower alpha diversity in disease farms, with the nasal microbiota from piglets from farms with disease by *M. hyorhinis* showing higher divergence from the healthy farms than those with Glässer’s disease. As it has been previously reported, imbalances in richness and/or diversity in different microbiome landscapes are associated with changes that generally may lead to disease in the host organism [15]. In swine production, changes in the nasal microbiota of the weaning piglets may influence the subsequent development of Glässer’s disease [6].

Antimicrobial usage is one of the plethora of factors that can affect the pig microbiome in commercial farms. The animals included in this study underwent different antimicrobial treatments, which may impact the nasal microbiota composition. However, we found that different farms under the same antimicrobial treatment but with different health status (GE, RM, GM) did not cluster together, suggesting that these treatments did not seem to be determinant in the subsequent health status of the animals. Although the present study presents some limitations due to the different factors affecting the microbiome composition of the animals in commercial farms, our results reinforce the general idea that lower alpha diversity is related with disease development [6,16]. Lower bacterial richness and evenness was found in the nasal microbiota in the farms with disease, when they were compared with the animals from the healthy control farms. This finding is supported by many other studies that established that lower alpha diversity values are linked to poor health status in pigs, but also in other species [13,16,17]. Here, a lower microbial richness of the nasal cavity in the disease farms may be associated with the proliferation of the undesirable taxonomic groups that would lead to systemic infections. However, the nasal microbiota composition from the two different disease groups differed, which indicates that different microbiota imbalances may predispose the animals to different infections. In general, the composition from the farms with Glässer’s disease were more similar to the healthy ones but different from the *M. hyorhinis* farms. At higher taxonomic levels (phylum to order), highly similar composition between the disease groups MH and GD was observed, while at lower levels (family to species) differences between these two disease groups were evident. In fact, the distribution of the most abundant ASVs showed a clear different pattern in MH and GD farms. Nevertheless, we were able to find some taxa associated with health, such as the *Prevotellaceae* family, which deserves further study. This family was also represented in the healthy farms at the ASVs level by *Prevotella copri* and *Prevotella stercorea*, two species previously found in higher abundance in suckling and weaning healthy piglets [18]. Two ASVs from the *Lachnospiraceae* family were also associated with healthy farms, in agreement with previous studies, where ASVs from this family were correlated with higher feed conversion ratio [19].

Animals from control farms showed a lower relative abundance of *M. hyorhinis* and *G. parasuis*. However, the relative abundance of the pathogens at the species level was not related to the posterior development of the corresponding systemic disease. Surprisingly, *G. parasuis* abundance was higher in MH farms, while *M. hyorhinis* was higher in GD farms. Although we cannot rule out a synergistic association between these two pathogens to develop disease, as it has been described before [20], our data seems to support the role of specific ASVs of each pathogen in disease development. It is well known that *G. parasuis* comprises strains of different pathogenic potential with different consequences for the piglet health [21,22]. In fact, colonization by virulent *G. parasuis* strains increases the risk of developing Glässer’s disease, while the non-virulent strains can provide some protection against the disease [22]. Although there are some suggestions in the literature of the existence of *M. hyorhinis* strains with different degree of virulence [23], and our data also support it, this has not been demonstrated. We detected specific ASVs of the pathogens that were found only in the disease farms and were not shared by the farms with different disease. The role of these specific *M. hyorhinis* ASVs in disease was supported by their detection in the clinical samples from those farms. Due to the limitations of the 16S rRNA gene amplicon sequencing, more studies with whole genome sequencing are needed. This result also reinforces the importance of study the microbiota at strain level, to better understand the intrinsic characteristics of the different strains and their role in the predisposition to the systemic infection.

In summary, different changes in the nasal microbiota composition were observed in weaning piglets from farms with polyserositis caused by either *M. hyorhinis* or *G. parasuis*. We hypothesize that these changes might facilitate dissemination and the subsequent development of the systemic infections by *M. hyorhinis* and *G. parasuis*. The strain level resolution of the microbiota should be studied for virulent-strain detection, especially in the case of *M. hyorhinis* where there is a lack of studies addressing this issue.

## 4. Materials and Methods

### 4.1. Farm Selection and Sampling

Farms were selected based on the presence/absence of post-weaning polyserositis cases to study the nasal microbiota. Three groups of farms were included in the study, (Table 1). The first group, MH, was formed by two farms where polyserositis cases in nursery pigs were ascribed to *M. hyorhinis*. For such a purpose, samples from polyarthritis and/or polyserositis lesions were taken from animals at necropsy and *M. hyorhinis* was detected by qPCR [24] and/or isolation. In addition, four of the positive clinical samples (2 from RM and 2 from GE farms) were submitted at Servei de Genòmica, Universitat Autònoma de Barcelona to sequence the 16S rRNA gene using Sanger technology. All the samples from necropsies from MH farms were negative to isolation and/or PCR of *G. parasuis* [25] and *S. suis* [26]. In each MH farm, nasal swabs were taken from ten 3–4 week-old piglets selected from different litters (two piglets per sow) in order to avoid a sow effect bias. The second group was formed by three control farms without polyserositis or respiratory cases in the last two years previous to the study (Group HC). The third group of farms, group GD, was composed of four farms in which the polyserositis problems were attributed to *G. parasuis*. Raw sequencing data from groups HC and GD belonged to a previous study (SRA Accession number SRP068182 [6]).

Sampling of piglets was done under institutional authorization and followed good veterinary practices. According to European (Directive 2010/63/EU) and Spanish (Real Decreto 53/2013) normative, this procedure did not require specific approval by an Ethical Committee (Chapter I, Article 1, 5 (f) of 2010/63/EU).

### 4.2. DNA Extraction and 16S rRNA Gene Amplicon Sequencing

Nasal swabs were placed in 500 μL de PBS, vortex for 30 s and stored at −80 °C until used for DNA extraction. DNA was extracted from 200 μL of the initial 500 μL PBS where the swabs were resuspended and eluted in 100 μL of PBS using the Nucleospin Blood (Macherey Nagel, Bethlehem, PA, USA) kit. Quantity and quality assessment of the DNA was performed using BioDrop DUO (BioDrop Ltd., Cambridge, UK). Samples were submitted for 16S rRNA gene amplicon sequencing using the Illumina paired-end 2 × 250 bp kit (MS-102-2003 MiSeq^®^ Reagent Kit v2, 500 cycle) following the manufacturer’s instructions. The library preparation for sequencing was performed within 24 h after the DNA extraction at Servei de Genòmica, Universitat Autònoma de Barcelona. The region amplified was V3–V4 that covers two hypervariable regions of the conserved gene, and it was sequenced according to the Illumina protocol [27] as previously described [6]. The entire sequence dataset is available at the NCBI database, SRA accession number PRJNA717778.

### 4.3. Microbiota In-Silico Analysis

The in-silico analysis was done using the plugin based software Quantitative Insights into Microbial Ecology (QIIME) vs 2020.11 [28]. First, after importing the raw sequencing data into Qiime2 (*q2-import*), we performed a quality check step and decided to trim the reads to a length of 240 bp based on the quality drop at the end of the reads. Denoising, trimming and quality-based filtering was performed with the *q2-dada2* plugin [29], which also includes a chimera detection and consequent removal. Moreover, to improve the quality of our dataset, unassigned taxa was filtered out after aligning the ASVs against the Greengenes reference database (vs. 13.8) [30], clustered at 88% identity with 65% of identity and over the 50% of query [31].

With the rarefied curves of the richness, we extracted the proper sample-depth as a key parameter for the following diversity calculations. Diversity within each sample (alpha diversity) was estimated with Shannon’s entropy index, Observed Features and Chao index using *q2-core-metrics* plugin [32,33]. To compare the microbiota composition among the study groups (beta diversity), we calculated Jaccard, Bray Curtis and Unifrac [34,35,36] (weighted and unweighted) metrics and represented in the spatial coordinates with the Principal Components Analysis (PCoA). PERMANOVA [37] tests were performed to analyze beta diversity clusters among study groups. Also, the Adonis function [38] from Vegan package [39] was used across the matrix distances in order to determine the percentage of explanation from the grouping variables analyzed.

Taxonomic assignment of the ASVs was performed by a pre-trained Naive Bayes classifier within the *q2-feature-classifier classify-sklearn* plugin [40] using the Greengenes database (Vs. 13.8) [30]. Classification of ASVs from *Glaesserella* and *Mycoplasma* genera was confirmed to species level using Basic Local Alignment Search Tool (Blast) [41] algorithm and NCBI RefSeq database [42]. To find differentially abundant features across groups, analysis of compositional microbiomes (ANCOM) [43] was performed. To process and analyze the data from the taxa assignment of the ASVs and the counts of the features we used qiime2r (18), phyloseq [44], tidyverse [45] R packages. Heatmap, PCoA and Venn diagrams plots were built in Rstudio [46] using ggplot2 [47], ComplexHeatmap [48] and ggVennDiagram [49] packages. The phylogenetic tree of the ASVs from *M. hyorhinis* and *G. parasuis* were built with Maximum likelihood algorithm using Qiime2 [28].

## Figures and Tables

**Table 1:** Main characteristics of the farms and number of samples included in the study.

Farm (n *)	Group	Health Status	Production System	Size **	Treatments ***	Reference
RM (10)	MH	Polyserositis by *M. hyorhinis*	Multi-site	650	Amx	This study
GE (10)	MH	Polyserositis by *M. hyorhinis*	Multi-site	800	Amx	This study
VL (5)	HC	Healthy	Farrow to finish	700	Tlt-Ceft	[6]
PT (5)	HC	Healthy	Multi-site	1000	NA	[6]
GM (10)	HC	Healthy	Multi-site	1200	Amx	[6]
MT (8)	GD	Glässer’s disease	Multi-site	3300	Pen-Strep	[6]
MC (10)	GD	Glässer’s disease	Farrow to finish	480	Ceft	[6]
RC (6)	GD	Glässer’s disease	Multi-site	1400	Ceft	[6]
EJ (10)	GD	Glässer’s disease	Multi-site	2000	Enro	[6]

* number of samples included from each farm; ** n. of sows; *** Perinatal antimicrobials: Amx, amoxicillin; Tlt, tulathromycin; Ceft, ceftiofur; Pen, Penicillin; Strep, streptomycin; Enro, enrofloxacin; NA, not available.

## Data Availability

The entire sequence dataset is available at the NCBI database, SRA accession number PRJNA717778.

## Supplementary Materials

The following are available online at https://www.mdpi.com/article/10.3390/pathogens10050603/s1, Figure S1: Beta diversity analysis on Jaccard (A) and weighted Unifrac distances (B) of the nasal microbiota of weaning piglets. Each circle represents the microbial composition of each sample and the size of the circles is proportional to the number of Observed Features as indicated in the legend. Healthy control farms (HC) are depicted in blue, while farms with polyserositis caused by *M. hyorhinis* (MH) are in orange and *G. parasuis* (GD) in green palettes. Ellipses are calculated with the euclidean distances of the grouped samples. Table S1: Differential abundance analysis (ANCOM) comparing the nasal microbiota of weaning piglets from the three different group of farms (GD, farms with Glässer’s disease; MH, farms with polyserositis caused by *M. hyorhinis* and HC, healthy control farms) at ASVs level. Different comparisons are shown in the different excel sheets as indicated in each sheet label. Table S2: Differential abundance analysis (ANCOM) comparing the nasal microbiota of weaning piglets from the three different group of farms (GD, farms with Glässer’s disease; MH, farms with polyserositis caused by *M. hyorhinis* and HC, healthy control farms) at phylum level. Different comparisons are shown in the different excel sheets as indicated in each sheet label. Table S3: Differential abundance analysis (ANCOM) comparing the nasal microbiota of weaning piglets from the three different group of farms (GD, farms with Glässer’s disease; MH, farms with polyserositis caused by *M. hyorhinis* and HC, healthy control farms) at class level. Different comparisons are shown in the different excel sheets as indicated in each sheet label. Table S4: Differential abundance analysis (ANCOM) comparing the nasal microbiota of weaning piglets from the three different group of farms (GD, farms with Glässer’s disease; MH, farms with polyserositis caused by *M. hyorhinis* and HC, healthy control farms) at order level. Different comparisons are shown in the different excel sheets as indicated in each sheet label. Table S5: Differential abundance analysis (ANCOM) comparing the nasal microbiota of weaning piglets from the three different group of farms (GD, farms with Glässer’s disease; MH, farms with polyserositis caused by *M. hyorhinis* and HC, healthy control farms) at family level. Different comparisons are shown in the different excel sheets as indicated in each sheet label. Table S6: Differential abundance analysis (ANCOM) comparing the nasal microbiota of weaning piglets from the three different group of farms (GD, farms with Glässer’s disease; MH, farms with polyserositis caused by *M. hyorhinis* and HC, healthy control farms) at genus level. Different comparisons are shown in the different excel sheets as indicated in each sheet label. Table S7: Differential abundance analysis (ANCOM) comparing the nasal microbiota of weaning piglets from the three different group of farms (GD, farms with Glässer’s disease; MH, farms with polyserositis caused by *M. hyorhinis* and HC, healthy control farms) at species level. Different comparisons are shown in the different excel sheets as indicated in each sheet label.
