# Supplementary figures and images for: Altered Nasal Microbiota Composition Associated with Development of Polyserositis by Mycoplasma hyorhinis
Source: Pathogens. 2021 May 14;10(5):603. doi: 10.3390/pathogens10050603 (PMC8156107; doi:10.3390/pathogens10050603)

**A**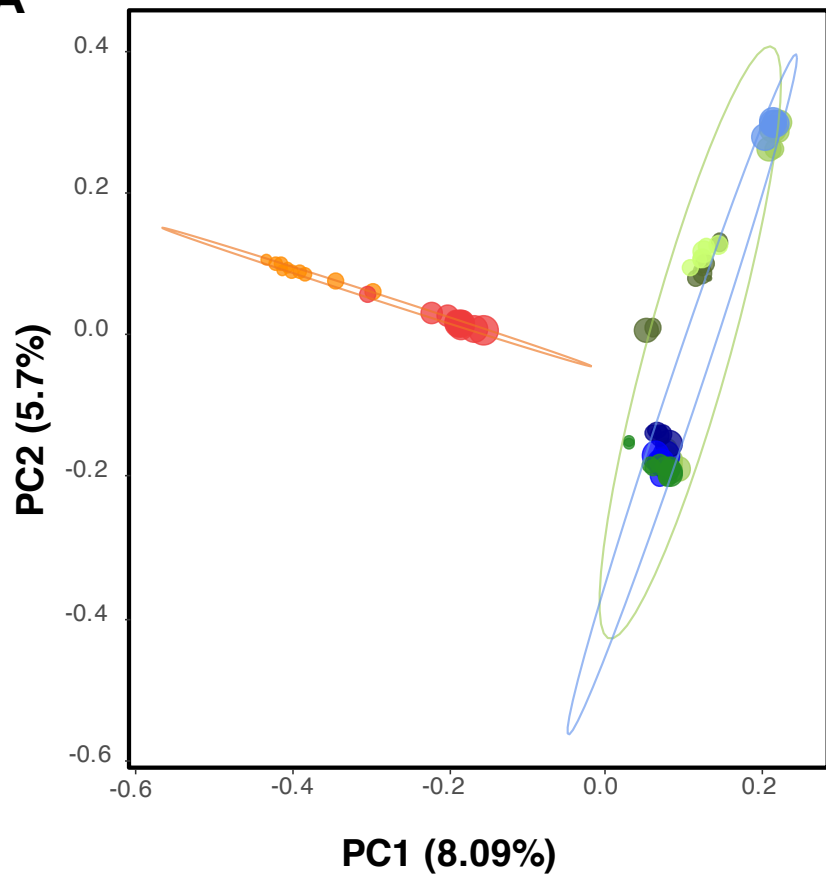**B**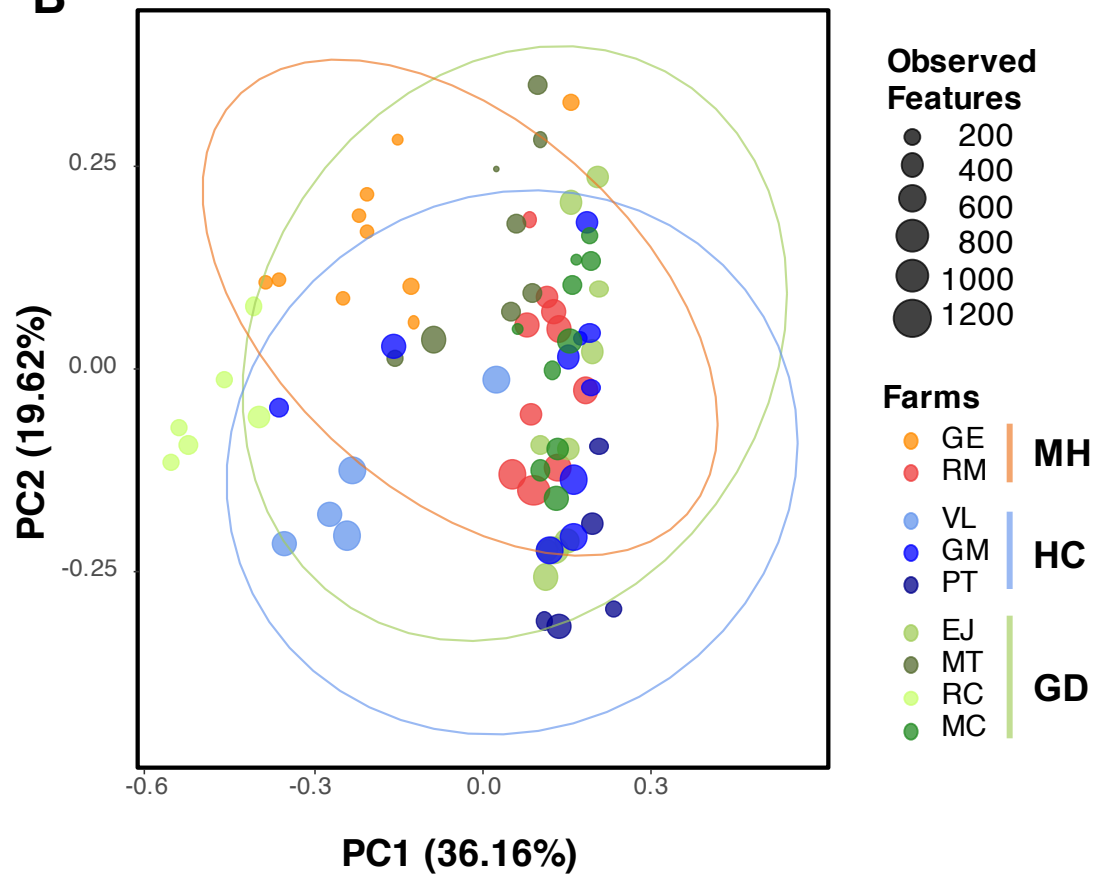

Supplement: Supplementary file 1 [file pathogens-10-00603-s001.zip › pathogens-1189127-supplementary/SupplementaryFiles/SupplementaryFigureS1.pdf]
